# Glucagon-Like Peptide 1 Receptor Agonists – Potential Game Changers in the Treatment of Glaucoma?

**DOI:** 10.3389/fnins.2022.824054

**Published:** 2022-02-21

**Authors:** Zaynab Ahmad Mouhammad, Rupali Vohra, Anna Horwitz, Anna-Sophie Thein, Jens Rovelt, Barbara Cvenkel, Pete A. Williams, Augusto Azuara-Blanco, Miriam Kolko

**Affiliations:** ^1^Department of Drug Design and Pharmacology, University of Copenhagen, Copenhagen, Denmark; ^2^Department of Veterinary and Animal Sciences, University of Copenhagen, Copenhagen, Denmark; ^3^Department of Ophthalmology, Copenhagen University Hospital, Rigshospitalet, Glostrup, Denmark; ^4^Department of Ophthalmology, University Medical Centre Ljubljana, Ljubljana, Slovenia; ^5^Medical Faculty, University of Ljubljana, Ljubljana, Slovenia; ^6^Division of Eye and Vision, Department of Clinical Neuroscience, St. Erik Eye Hospital, Karolinska Institutet, Stockholm, Sweden; ^7^Centre for Public Health, Queen’s University Belfast, Belfast, United Kingdom

**Keywords:** glaucoma, GLP-1 receptor agonists, antidiabtics, ophthalmology, neuroprotection, neurodegenerative diseases

## Abstract

Glaucoma is a common ocular neurodegenerative disease characterized by the progressive loss of retinal ganglion cells and their axons. It is the most common cause of irreversible blindness. With an increasing number of glaucoma patients and disease progression despite treatment, it is paramount to develop new and effective therapeutics. Emerging new candidates are the receptor agonists of the incretin hormone glucagon-like-peptide-1 (GLP-1), originally used for the treatment of diabetes. GLP-1 receptor (GLP-1R) agonists have shown neuroprotective effects in preclinical and clinical studies on neurodegenerative diseases in both the brain (e.g., Alzheimer’s disease, Parkinson’s disease, stroke and diabetic neuropathy) and the eye (e.g., diabetic retinopathy and AMD). However, there are currently very few studies investigating the protective effects of GLP-1R agonists in the treatment of specifically glaucoma. Based on a literature search on PubMed, the Cochrane Library, and ClinicalTrials.gov, this review aims to summarize current clinical literature on GLP-1 receptor agonists in the treatment of neurodegenerative diseases to elucidate their potential in future anti-glaucomatous treatment strategies.

## Introduction

Glaucoma is one of the most common neurodegenerative eye diseases and the leading cause of irreversible blindness. It is estimated to affect ∼112 million people worldwide by 2040 (Tham et al., 2014). Glaucoma is characterized by the progressive and irreversible loss of retinal ganglion cells, the output neurons of the retina. There are three primary risk factors for glaucoma; increasing age, genetic risk and elevated intraocular pressure (IOP). Elevated IOP is strongly associated with disease progression in ∼60% of patients, but all available anti-glaucomatous therapies (pharmacological and surgical) exclusively target IOP (Kolko, 2015, 2017; Kolko et al., 2015; Kalouda et al., 2017; Quigley, 2019). The number of glaucoma cases associated with an actually elevated IOP varies significantly across glaucoma subtypes, and many glaucoma patients progress to blindness despite low IOPs and/or are refractory to IOP-lowering treatments (Dielemans et al., 1994; Heijl et al., 2002; Topouzis and Anastasopoulos, 2007; Cedrone et al., 2008; Peters et al., 2013; Ho and Wong, 2019). Therefore, there is an urgent unmet need to explore new therapies targeting mechanisms of glaucomatous neurodegeneration in addition to IOP reduction. One such important new route of study in glaucoma, and the focus of this review, may be the glucagon-like peptide 1 (GLP-1).

Glucagon-like peptide 1 (GLP-1) is an incretin hormone that, like its fellow incretin hormone GIP (glucose-dependent insulinotropic polypeptide), stimulates a decrease in blood glucose levels after nutrient intake. GLP-1 achieves the decrease in blood glucose levels by potentiating glucose-induced insulin secretion (the “incretin”-effect), improving insulin sensitivity and inhibiting glucagon release. Furthermore, GLP-1 regulates weight and satiety and delays gastric emptying (Gutniak et al., 1992; Drucker, 2018; Yildirim Simsir et al., 2018). The first agents targeting GLP-1 receptor (GLP-1R) signaling were developed to optimize the treatment of type 2 diabetes mellitus (T2D) as an adjunct to metformin when therapeutic goals where not met (Yildirim Simsir et al., 2018). The first GLP-1R agonists received FDA-approval in 2005 and included *exenatide.* Subsequently, many different GLP-1R agonists were developed and approved, including*: extended release exenatide (exenatide XR), liraglutide, lixisenatide*, *dulaglutide*, and *semaglutide* (Table 1). Although all GLP-1R agonists are effective in stabilizing blood glucose levels, glycated hemoglobin (HbA1c), cholesterol levels, insulin sensitivity, and several other beneficial aspects of metabolism in T2D patients (Courrèges et al., 2008; Patel et al., 2014; Jinnouchi et al., 2015; Rizvi et al., 2015; Rizzo et al., 2015, 2016), drug administration has been challenging. Until recently, the treatments had to be administered subcutaneously (Table 1). In September 2019, the first oral GLP-1R agonist for T2D was approved by FDA (Davies et al., 2017; Aroda et al., 2019; Husain et al., 2019; Mosenzon et al., 2019; Pieber et al., 2019; Pratley et al., 2019; Rodbard et al., 2019; Rosenstock et al., 2019; The U.S. Food and Drug administration, 2019; Zinman et al., 2019a; Yabe et al., 2020; Yamada et al., 2020; Khoo and Lin, 2021). With its great beneficial systemic effects and promising results from previous randomized clinical trials with injectable semaglutide, the oral GLP-1R agonist, Rybelsus^®^, also seem to be a promising and convenient anti-obesity treatment option (Khoo and Lin, 2021).

**TABLE 1:** Comparison of various currently available GLP-1 receptor agonists.

Drug *(Commercial name)*	Administration		HbA1c	Weight	GI-adverse effects	Patient persistence	Preclinically proven neuro-protective effect
Exenatide *(Byetta ^®^)*	*s.c.*	*Twice daily*	Low	Low	Highest	−	Yes
Exenatide XR *(Bydureon ^®^)*	*s.c.*	*Once weekly*	Intermediate	Low	low	low	
Lixisenatide *(Lyxumia ^®^/* *Adlyxin ^®^)*	*s.c.*	*Once daily*	Low	Low	Intermediate	−	Yes
Liraglutide *(Victoza ^®^)*	*s.c.*	*Once daily*	High	High	Intermediate/high	Intermediate	Yes
Dulaglutide *(Trulicity ^®^)*	*s.c.*	*Once weekly*	High	Intermediate	Intermediate/high	High	Yes
Semaglutide *(Ozempic ^®^)*	*s.c.*	*Once weekly*	Highest	Highest	High	High	Yes
Semaglutide *(Rybelsus ^®^)*	*p.o.*	*Once daily*	Highest/ high	Highest	Intermediate/high	Highest*	

*Based on results from HbA1c-levels and bodyweight reductions of patients, semaglutide seem to be the most efficient GLP-1R agonist. However, semaglutide may also cause higher rates of gastrointestinal (GI) adverse effects. In general, once weekly or orally administrated GLP-1R agonists, i.e., oral semaglutide, are preferred amongst patients. The scheme is inspired by Trujillo et al. (2021) GLP-1 receptor agonists: an updated review of head-to-head clinical studies and patients’ persistence of treatment based on estimations by Uzoigwe et al. (2021) Semaglutide Once-Weekly Persistence and Adherence Versus Other GLP-1 RAs in Patients with Type 2 Diabetes in a US Real-World Setting. P.o.; per oral, s.c.; subcutaneous.*

*Based on a Japanese diabetes treatment related quality of life questionnaire oral semaglutide seemed to be preferred over injectable dulaglutide (i.e., injectable GLP-1R agonist) by the participating patients (Ishii et al., 2021).

Glucagon-like peptide 1 (GLP-1) agonists not only exert a beneficial systemic effect, but also cross the blood-brain barrier (Kastin et al., 2002; Secher et al., 2014; Yildirim Simsir et al., 2018). In this context, GLP-1R agonists have been shown to affect the central nervous system and exhibit neuroprotective properties in animal models of several neurodegenerative diseases, including Alzheimer’s and Parkinson’s disease, stroke, diabetic retinopathy, and ocular hypertension (McClean et al., 2011; Hao et al., 2012; Fan et al., 2014; Gonçalves et al., 2016; Zhang et al., 2018, 2019; Basalay et al., 2019; Yang et al., 2019; Chang et al., 2020; Ramos et al., 2020; Ren et al., 2020; Sterling et al., 2020; Zhai et al., 2020). The potential neuroprotective properties of GLP-1R agonists have also been demonstrated in clinical trials, with Novo Nordisk currently running a phase III clinical trial of oral semaglutide as a potential treatment option for Alzheimer’s disease in the announced EVOKE trial program (NCT04777409, NCT04777396).

In addition to GLP-1R agonists, orally administrated DPP4-inhibitors, e.g., *sitagliptin* and *saxagliptin*, which are known to potentiate the effect of endogenous GLP-1 by inhibiting its degradation, have also been clinically studied as protective agents against retinopathy (Chung et al., 2016), Alzheimer’s disease (Isik et al., 2017), and Parkinson’s disease (Svenningsson et al., 2016) in T2D patients, although DPP4-inhibitors cannot cross the blood-brain barrier (Mousa and Ayoub, 2019).

Recently, a registry-based case-control study of 1,961 patients also associated the use of GLP-1R agonists with a reduced risk of glaucoma (Sterling et al., 2021), strongly supporting further research into the use of agents that increase GLP-1R signaling as anti-glaucomatous treatment strategies. Accordingly, previous studies of oral antidiabetics and insulin have also been proposed for the treatment of glaucoma as well as other ocular conditions such as age-related macular degeneration (AMD) (Ott et al., 2014; Lin et al., 2015, 2017; Chung et al., 2016, 2019; Maleškić et al., 2017; Douros et al., 2018; Kim et al., 2018; Li et al., 2018; Vilsbøll et al., 2018; Wang et al., 2018; Brown et al., 2019; Chen et al., 2019; Fan et al., 2020; Gaborit et al., 2020; Blitzer et al., 2021; Sterling et al., 2021). The present review aims to summarize current literature on antidiabetics in the treatment of neurodegenerative diseases with a special focus on GLP-1R agonists and glaucoma.

## Method of Literature Search

**FIGURE 1:**
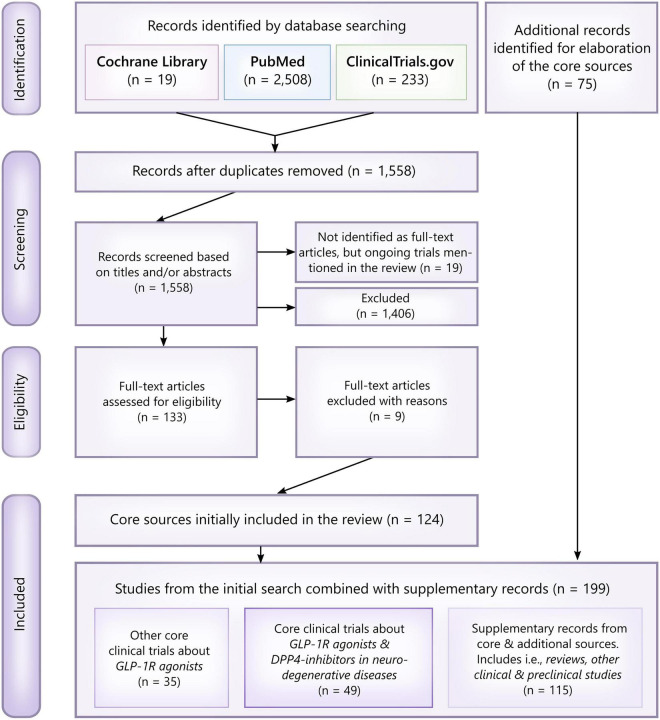
PRISMA flowchart and overview of the literature search. Studies were primarily excluded based on their clinical relevance and whether or not they assessed a neuroprotective effect of GLP-1R agonists/DPP4-inhibitors or not. Thus, included core studies were assessed as eligible if identified as randomized clinical trials, pilot studies, other interventional studies, epidemiological studies (cohort studies, case-control studies, etc.) and studies analyzing samples from clinical trials. For elaboration of the core sources, i.e., reviews, preclinical studies investigating the neuroprotective mechanisms behind GLP-1R agonists, relevant abstracts and *post hoc* analyses were included as additional records. In total, 199 records were included.

PubMed and the Cochrane Library were searched in April 2021 (Figure 1). The search was not limited to a specific language. However, mainly clinical studies and clinically inclined reviews were included as core sources. In addition, the Cochrane Library was screened for relevant Cochrane reviews only. MeSH-controlled vocabulary terms and related keywords were combined using appropriate Boolean operators to find literature either investigating or discussing incretin-based drugs (GLP-1R agonists and DPP4-inhibitors) for the treatment of neurodegenerative diseases, particularly glaucoma, diabetic retinopathy, AMD, Alzheimer’s disease, Parkinson’s disease, stroke and neuropathy. Search words included: *GLP-1, Glucagon-Like Peptide 1, semaglutide, oral semaglutide, Rybelsus, Ozempic, liraglutide, lixisenatide, dulaglutide, albiglutide, exenatide, DPP4 inhibitor, dipeptidyl phosphatase 4 inhibitor, sitagliptin, antidiabetics, metformin, insulin, type 2 diabetes, diabetes, diabetes mellitus, neurodegenerative diseases, glaucoma, AMD, age-related macular degeneration, diabetic retinopathy, elderly, Alzheimer, dementia, cognitive impairment, Alzheimer’s Disease, Parkinson, Parkinson’s Disease, stroke, neuropathy*. The reference lists of all core sources were searched for additional references. Completed and ongoing trials examining the role of GLP-1R agonists, especially semaglutide, in the treatment of glaucoma, AMD, Alzheimer’s, and Parkinson’s disease, were searched on the public clinical trial registry clinicaltrials.gov (*NCT04777409, NCT04777396, NCT03811561, NCT01843075, NCT04232969, NCT03659682, NCT02953665, NCT04305002, NCT03439943, NCT04269642, NCT04154072, NCT02673931, NCT02829502, NCT02838589, NCT00418288, NCT00256256, NCT03948347, NCT03287076*, *NCT02684578, NCT05035095*). Completed clinical trials with unknown status, no recent updates, no public or published results, and no relevance to the subject of the current review were excluded.

## Results and Discussion

### Oral Semaglutide Has Great Potential for the Prevention of Retinal Neurodegeneration

**FIGURE 2:**
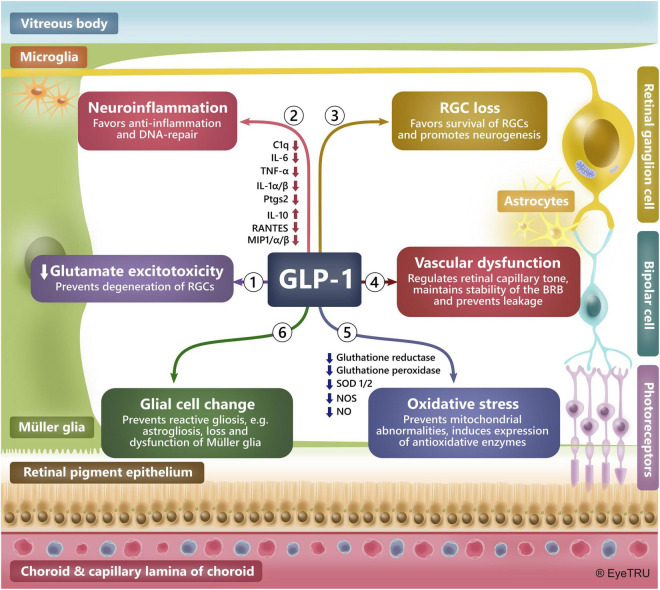
Mechanisms behind a neuroprotective effect of GLP-1R agonists in the retina. Preclinical studies have demonstrated a glia- and neuroprotective effect of GLP-1R agonists in the brain and retina. In the retina GLP-1R agonists have shown to protect against neurodegeneration by preventing: **(1) Glutamate excitotoxicity** by, e.g., upregulating glutamate transporters, **(2)** **Neuroinflammation** by, e.g., inducing anti-inflammatory cytokines, reducing levels of pro-inflammatory factors and favoring DNA-repair, **(3) Loss of retinal ganglion cells (RGCs)**, **(4) Vascular dysfunction** by, e.g., maintaining the blood-retinal barrier and regulating the tone of retinal capillaries, **(5) Oxidative stress** by, e.g., inducing an anti-oxidative environment and maintaining mitochondrial integrity, **(6)** **Glial cell change** by, e.g., reducing ocular hypertension-induced astrocyte reactivity, dysfunction and loss of Müller glia. *BRB: Blood-retina-barrier.*

There is a demonstrated neuroprotective effect of GLP-1R agonists in both the retina and brain in preclinical studies of ocular hypertension, diabetic retinopathy, Alzheimer’s disease, Parkinson’s disease, stroke, neuropathy, and several other neurodegenerative diseases (Kastin et al., 2002; McClean et al., 2011; Hao et al., 2012; Fan et al., 2014; Secher et al., 2014; Gonçalves et al., 2016; Zhang et al., 2018, 2019; Basalay et al., 2019; Yang et al., 2019; Chang et al., 2020; Ramos et al., 2020; Ren et al., 2020; Sterling et al., 2020; Zhai et al., 2020). In the retina, GLP-1R agonists have specifically protected retinal ganglion and glia cells, such as the Müller glia, from various stresses (Hao et al., 2012; Fan et al., 2014; Gonçalves et al., 2016; Yang et al., 2019; Ramos et al., 2020; Ren et al., 2020; Sterling et al., 2020) (Figure 2).

One of the most promising and effective GLP-1R agonists is *semaglutide* (Tables 1, 2) (Trujillo et al., 2021), which until September 2019 was only available as an injectable formulation. Semaglutide is also being tested in trials against diabetic eye diseases expected to end in 2027 (NCT03811561; *the FOCUS trial*). Now, with its ability to be administered orally, semaglutide offers great opportunities in the treatment of T2D as well as other potential disease groups. Semaglutide was designed from the backbone of another GLP-1R agonist, *liraglutide*, which has been tested as a prospective neuroprotective treatment option against dementia (Femminella et al., 2019; Evaluate, 2020) (NCT01843075, *the ELAD study*). In addition, semaglutide; like dulaglutide and exenatide XR, were developed to act as a long-acting GLP-1 analog, replacing the previous subcutaneous injections once daily with once weekly administration, potentially improving patient adherence and convenience (Lau et al., 2015).

**TABLE 2:** Clinical interventional studies elucidating the efficacy and safety-profile of oral semaglutide in comparison to placebo or other antidiabetics in patients with T2D.

Comparator treatment		Study phase and name	Study design	Results	References
Placebo	Placebo	*Phase: III* *PIONEER 1* *PIONEER 4* *PIONEER 5* *PIONEER 6* *PIONEER 8* *PIONEER 9*	5,895 patients with T2D were randomly assigned to receive daily oral semaglutide (3, 7 or 14 mg/day) or placebo for 26 or 52 weeks. Patients were either medication-naïve or medicated with other second-line antidiabetics.	Superiorly reduced HbA1c levels at all doses and mean body weight. Did not change the renal function of participants and had a non-inferior cardiovascular safety profile compared to placebo. Also, semaglutide allowed participants using basal insulin to reduce their daily insulin doses by 15-25%. Gastrointestinal events, mainly mild-to-moderate nausea, were more common with oral semaglutide than with placebo.	Aroda et al., 2019; Husain et al., 2019; Mosenzon et al., 2019; Pratley et al., 2019; Zinman et al., 2019a; Yamada et al., 2020
GLP-1R agonists	Injectable semaglutide	*Phase: II*	632 patients with T2D were randomized to receive *oral semaglutide* (2.5, 5, 10, 20 mg/day or escalation to 40 mg/day), *subcutaneous semaglutide* (1.0mg/week) or placebo for 26 weeks.	Improved HbA1c and mean body weight non-inferiorly compared to subcutaneous semaglutide.	Davies et al., 2017
	Liraglutide *or* dulaglutide	*Phase: II/III* *PIONEER 4* *PIONEER 9* *PIONEER 10*	1,412 patients with T2D were randomly assigned to receive *oral semaglutide* (3, 7 or 14 mg/day), *subcutaneous dulaglutide* (0.75 mg/week) or *liraglutide* (0.9 mg/week or escalation to 1.8 mg/day) for 52 or 57 weeks. Patients were either medication-naïve or medicated with other antidiabetics.	Superiorly or non-inferiorly reduced HbA1c levels and mean body weight. A dose of 14 mg/day was especially efficient.	Pratley et al., 2019; Yabe et al., 2020; Yamada et al., 2020
Other antidiabetics	Sitagliptin *or* empagliflozin	*Phase: III* *PIONEER 2* *PIONEER 3* *PIONEER 7*	3,189 patients with T2D were randomly assigned to receive daily *oral semaglutide* (3, 7 or 14 mg/day), *sitagliptin* (100mg/day) or *empagliflozin* (25 mg/day) for 52 weeks or up to 78 weeks.	Superiorly reduced HbA1c levels and mean body weight. Doses of 7 and 14 mg/day were especially efficient.	Pieber et al., 2019; Rodbard et al., 2019; Rosenstock et al., 2019

*Compared to other antidiabetic drugs, including subcutaneously administrated GLP-1R agonists, oral semaglutide is associated with significant improvements in HbA1c levels and body weight of patients with T2D and is either non-inferior or superior in effect.*

The safety and efficacy of semaglutide have been assessed in several phase II and III clinical trials (Marso et al., 2016a; Nauck et al., 2016; Ahrén et al., 2017; Aroda et al., 2017, 2019; Davies et al., 2017; Sorli et al., 2017; Ahmann et al., 2018; Kaku et al., 2018; Pratley et al., 2018, 2019; Rodbard et al., 2018, 2019; Seino et al., 2018; Husain et al., 2019; Lingvay et al., 2019; Mosenzon et al., 2019; Pieber et al., 2019; Rosenstock et al., 2019; Zinman et al., 2019a,b; Capehorn et al., 2020; Ji et al., 2020; Yabe et al., 2020; Yamada et al., 2020). Among these, there are 15 studies assessing the injectable semaglutide as part of the SUSTAIN trial series and 10 evaluating the newly approved oral semaglutide as part of the PIONEER trial program (Table 2). Through the SUSTAIN 1-10 (Marso et al., 2016a; Ahrén et al., 2017; Aroda et al., 2017; Sorli et al., 2017; Ahmann et al., 2018; Pratley et al., 2018; Rodbard et al., 2018; Lingvay et al., 2019; Zinman et al., 2019b; Capehorn et al., 2020) trials, the effect of injectable semaglutide, measured as reductions in glycated hemoglobin and bodyweight, was generally significantly better than treatment with DPP4-inhibitors (*sitagliptin*) (Ahrén et al., 2017; Seino et al., 2018; Ji et al., 2020), SGLT-2-inhibitors (*canagliflozin*) (Lingvay et al., 2019), long-acting insulin (*insulin glargine*) (Aroda et al., 2017), and other GLP-1R agonists [*exenatide XR* (Ahmann et al., 2018), *dulaglutide* (Pratley et al., 2018) and *liraglutide* (Capehorn et al., 2020)]. In the PIONEER 3 (Rosenstock et al., 2019) and 7 (Pieber et al., 2019) trials, *oral* semaglutide was also found to be superior to the DPP4-inhibitor sitagliptin. In addition, a retrospective database study estimated that patients’ persistence of injectable semaglutide treatment was significantly higher than treatment with dulaglutide, liraglutide and exenatide XR (Table 1) (Uzoigwe et al., 2021). Semaglutide has also shown promise as an effective anti-obesity treatment option. In a randomized clinical trial, a dose of 2.4 mg injectable semaglutide weekly in subjects with or without weight-related complication, semaglutide, along with lifestyle interventions, was associated with sustained as well as clinically relevant reduction in body weight (Khoo and Lin, 2021). In 2021, Novo Nordisk launched the OASIS trial expected to end in 2023 assessing the beneficial effects and safety-profile of 50 mg daily semaglutide compared to placebo (NCT05035095).

When comparing oral semaglutide with injectable GLP-1R agonists as in the PIONEER 4 trial (Pratley et al., 2019), oral semaglutide appears to be an even better option than injectable alternatives. In addition, daily oral semaglutide was found to be ranked as the best GLP-1R agonist to reduce cardiovascular death and death from any cause when indirectly compared with injectable *semaglutide, liraglutide, lixisenatide, dulaglutide, albiglutide, exenatide* and placebo (Alfayez et al., 2020). Compared to subcutaneously injected semaglutide, a phase II trial showed that oral semaglutide was equally effective in improving HbA1c and body weight for the T2D patients included in the trial (Davies et al., 2017).

Oral semaglutide is also likely to be more cost-effective than other existing GLP-1R agonists in the treatment of T2D patients (Hansen et al., 2020). Across the PIONEER trials, the most common adverse effects of oral semaglutide were related to the gastrointestinal tract, similar to the injectable GLP-1R agonists, and manifested as: vomiting, diarrhea and nausea (Seidu et al., 2021). In addition, oral semaglutide was not associated with severe cardiovascular outcomes or further kidney impairment in patients with renal dysfunction (Mosenzon et al., 2019). This further supports oral semaglutide as effective despite long-standing T2D and comorbidities. Thus, oral semaglutide appears to be the most effective and convenient option compared to other GLP-1R agonists, DPP4-inhibitors, and second-line antidiabetic agents. Novo Nordisk also recently started recruiting participants to the EVOKE trial program (phase III) expected to end in 2024, in which the effectiveness of oral semaglutide in the treatment of early Alzheimer’s disease will be assessed by measuring changes in clinical dementia rating, daily activity, mild cognitive impairment and mini-mental state examinations and other secondary outcome measures (NCT04777409, NCT04777396). Furthermore, as semaglutide is the only GLP-1R agonist that can be administrated as both an oral and injectable formulation, the selection of the most convenient mode of administration can be made on an individual level to best suit the patients’ preferences and needs. Given the potential neuroprotective and insulin sensitivity-regulating effects of compounds that increase GLP-1R signaling, semaglutide appears to be a promising candidate for further investigation as a novel add-on compound in the treatment of neurodegenerative diseases, such as glaucoma.

#### Glucagon-Like Peptide 1 Agonists and Other Antidiabetic Agents as a Potential Treatment for Glaucoma

Several antidiabetic compounds have shown potential for glaucoma treatment (Lin et al., 2015; Maleškić et al., 2017; Sterling et al., 2021) (Table 3). In support of this, several studies have suggested that diabetes mellitus is a risk factor for glaucoma (Oshitari et al., 2007; Chopra et al., 2008; Welinder et al., 2009; Zhou et al., 2014; Horwitz et al., 2016; Jung et al., 2018; Hanyuda et al., 2020). Thus, it raises the question of whether glaucoma may be associated with retinal insulin desensitization (Agostinone et al., 2018) as well as insulin desensitization of the brain as associated with Alzheimer’s and Parkinson’s disease (Faiq et al., 2014; Dada, 2017; Faiq and Dada, 2017). However, the mechanistic link and relationship between glaucoma and T2D still needs further analysis and requires further elucidation (Tielsch et al., 1995; Ellis et al., 2000; Quigley, 2009).

**TABLE 3:** Clinical studies elucidating the use of antidiabetics against glaucoma.

Compound		Study types	Study design	Study outcome	References
Glaucoma	GLP-1R agonists	Observational	1,961 patients with no baseline glaucoma, glaucoma suspect nor ocular hypertension who newly initiated GLP-1R agonist treatment, e.g., *semaglutide*, were compared to an unexposed control group.	Reduced the hazard for both a new diagnosis of glaucoma and glaucoma suspect (i.e., angle closure, no damage).	Sterling et al., 2021
	Metformin	Observational	150,250 patients with diabetes mellitus treated with *metformin* or other antidiabetic agents were followed and assessed.	Reduced the risk of developing glaucoma and other ocular complications as DR.	Lin et al., 2015; Maleškić et al., 2017

*Clinical studies elucidating the effects of GLP-1R agonists and metformin on the development of glaucoma. The use of antidiabetics is associated with a reduced risk of glaucoma.*

##### Metformin

Studies conducted by Lin et al. (2015) have shown that patients with T2D had a reduced risk of developing glaucoma when exposed to antihyperglycemic agents, in particular metformin, which was associated with a reduced risk of developing open-angle glaucoma. A later observational study also associated the use of metformin with a reduced risk of both glaucoma and diabetic retinopathy (Maleškić et al., 2017). The use of metformin has also been linked with a lower risk of developing other neurodegenerative eye conditions, such as AMD (Brown et al., 2019; Chen et al., 2019; Blitzer et al., 2021), and diabetic retinopathy (Maleškić et al., 2017; Li et al., 2018; Fan et al., 2020) (Table 4). The effects of metformin on AMD progression have not yet been directly investigated. However, there is currently an ongoing trial (scheduled for completion in 2023) that assesses the use of metformin to minimize geographic atrophy progression in AMD patients (NCT02684578).

**TABLE 4:** Clinical studies elucidating the use of antidiabetics against ocular conditions other than glaucoma.

Compound		Study types	Study design	Study outcome	References
AMD	Metformin	Observational	320,192 patients with or without AMD exposed/unexposed to metformin were compared.	Reduced odds of developing AMD (wet AMD, dry AMD and macular degeneration involving, e.g., drusen, retinal hemorrhaging or edema) among patients using metformin.	Brown et al., 2019; Blitzer et al., 2021
		Observational	68,205 patients with new-onset T2D using or not using metformin were assessed.	Lowered the risk of developing AMD among diabetic patients.	Chen et al., 2019
Diabetic Retinopathy	Metformin	Observational	10,379 patients with newly diagnosed or longstanding T2D (≥ 15 years) and DR using metformin or metformin along with DPP4-inhibitors or sulfonylurea were assessed.	Lowered the risk of severe non-proliferative, proliferative and sight-threatening DR. *DPP4-inhibitors* further reduced, and *sulfonylurea* increased the risk of DR.	Li et al., 2018; Fan et al., 2020
	Incretin-based therapies*	Observational	213,652 patients using incretin-based therapies were compared to patients using other second line antidiabetics, i.e., *sulfonylurea* and *long-acting insulin.* All patients had received no prior treatment for retinopathy.	Did not increase the risk of DR compared to other antidiabetics.	Wang et al., 2018
	GLP-1R agonists** &metformin	Observational	80,269 patients with T2D exposed to GLP-1R agonists were compared to patients treated with other antidiabetics or no add-on to metformin and insulin.	*GLP-1R agonist* exposure was not associated with severe DR, *metformin* was protective, and *insulin* strongly associated with severe DR. GLP-1R agonists were also found to decrease the risk of diabetic retinopathy.	Douros et al., 2018; Gaborit et al., 2020
	Liraglutide**	Phase IV interventional	50 patients with T2D and obesity were randomized to be treated for 4 weeks with 1.2 mg daily subcutaneous injections of liraglutide. Purpose was to assess levels of angiogenic biomarkers and hematopoietic progenitor cells associated with DR.	Was not associated with severe DR. Did not cause any significant differences in biomarkers and hematopoetic cells between treated and control group.	Gaborit et al., 2020
	Semaglutide	*Post hoc* analysis	*Post hoc* analyses of data on DR across the SUSTAIN clinical trial program (8.105 patients in total).	Did not directly cause early DR complications upon treatment initiation.	Vilsbøll et al., 2018
	DPP4-inhibitors***	Observational	11,282 patients with DR and/or only T2D using DPP4-inhibitors or other hypoglycemic agents with/without metformin were retrospectively reviewed and compared.	Did not increase the risk for DR and independently protected against the progression of DR.	Chung et al., 2016, 2019
	Sitagliptin	Observational	14,552 patients with T2D using DPP4-inhibitors, i.a. sitagliptin (11.026 patients), were followed and assessed for DR events.	Did not increase the overall risk of DR. However, short exposure and low cumulative doses were linked to greater risk of DR events.	Kim et al., 2018
	Saxagliptin	Phase III interventional	50 patients with T2D without DR were randomly assigned to receive either 5 mg saxagliptin or placebo for 6 weeks.	Reduced the retinal capillary blood flow and increased the vasodilatory capacity two-fold.	Ott et al., 2014
Other	Metformin	Observational	44,609 patients with T2D with no baseline retinal vein occlusion were followed and compared to non-diabetic subjects.	Protected against the development of retinal vein occlusion.	Lin et al., 2017

*Clinical studies elucidating the effects of antidiabetic agents, including GLP-1R agonists, DPP4-inhibitors and metformin, on the development and progression of ocular conditions. The use of antidiabetics is associated with a reduced risk of diabetic retinopathy (DR) and age-related macular degeneration (AMD). T2D; Type 2 diabetes.*

*Incretin-based therapies include: DPP4-inhibitors and GLP-1R agonists.

**The clinical part of the AngioSafe 1 T2D study includes an observational study, where exposure to, i.e., any GLP-1R agonist was assessed, and an interventional study, where the exposure to GLP-1R agonists only included liraglutide.

***DPP4-inhibitors included: vildagliptin, sitagliptin, saxagliptin, linagliptin or gemigliptin.

##### DPP4-Inhibitors

In addition, with respect to other ocular conditions and the use of antidiabetic drugs, DPP4-inhibitors have been suggested to exert a protective role in the progression of diabetic retinopathy (Ott et al., 2014; Chung et al., 2016, 2019; Kim et al., 2018). In a cohort study, DPP4-inhibitors appeared to protect against diabetic retinopathy progression, independent of their glucose-lowering effect (Chung et al., 2016). Another population-based study in South Korea comparing the use of DPP4-inhibitors with other oral glucose lowering agents (*sulfonylurea, thiazolidinedione*, and *metformin*) showed that the overall risk of diabetic retinopathy events was not increased (Kim et al., 2018). These findings are supported by a small double-blinded, placebo-controlled trial in 50 T2D patients, where 6 weeks of treatment with the DPP4-inhibitor, *saxagliptin*, increased vasodilation capacity and decreased retinal capillary blood flow (Ott et al., 2014). Another recent cohort study showed that the combination of metformin with DPP4-inhibitors had a strong beneficial effect against non-proliferative diabetic retinopathy (NPDR) (Fan et al., 2020). Moreover, the same study showed that the use of *sulfonylurea* instead of DPP4-inhibitors in combination with metformin increased the risk of NPDR (Fan et al., 2020). Thus, several clinical studies have linked the use of DPP4-inhibitors to either a reduced odds of diabetic retinopathy or a reduced risk of diabetic retinopathy progression (Ott et al., 2014; Kim et al., 2018; Wang et al., 2018; Chung et al., 2019; Fan et al., 2020).

##### Glucagon-Like Peptide 1 Agonists

Sterling et al. (2021) linked the exposure to GLP-1R agonists (*i.e., dulaglutide, liraglutide, lixisenatide, exenatide*, *and semaglutide*) to a low risk of developing open-angle glaucoma (Sterling et al., 2021). However, previous studies have raised concern that treatment with GLP-1R agonists may increase the risk of diabetic retinopathy, making them less favorable to be used against diabetic eye complications (Diabetes Control and Complications Trial Research Group, Nathan et al., 1993; No authors listed, 1998, 1999; Aiello and Dcct/Edic Research Group, 2014; Green et al., 2015; Marso et al., 2016b; Jingi et al., 2017; Tang et al., 2018; Bain et al., 2019; Lim et al., 2019; Bethel et al., 2021). Nevertheless, this effect of GLP-1R agonists is most likely caused by a rapid decrease in patients’ blood glucose levels as discussed later (Vilsbøll et al., 2018; Bain et al., 2019; Lim et al., 2019). The AngioSafe 1 study, which is also designed to clarify the association between exposure to GLP-1R agonists and diabetic retinopathy through clinical and preclinical study designs found no risk for severe diabetic retinopathy when patients were treated with GLP-1R agonists, and no effect of GLP-1R agonists on angiogenesis (Gaborit et al., 2020). In prospect, the effect of GLP-1R agonist exposure on severe diabetic retinopathy should be further elucidated in the ongoing AngioSafe 2 study (NCT02671864). Another phase III interventional study by Novo Nordisk, the FOCUS trial, also investigates the long-term effects of injectable semaglutide in diabetic eye diseases (NCT03811561).

#### Diabetic Retinopathy and Glucagon-Like Peptide 1 Agonists

Previous studies, including the DCCT, SUSTAIN-6, LEADER and TECOS trials, have linked exposure to incretin-based therapies and other hypoglycemic agents (i.e., *insulin*, *injectable semaglutide, liraglutide*, and *oral sitagliptin*) to the exacerbation of pre-existing retinopathy (Diabetes Control and Complications Trial Research Group, Nathan et al., 1993; No authors listed, 1998, 1999; Aiello and Dcct/Edic Research Group, 2014; Green et al., 2015; Marso et al., 2016a,b; Jingi et al., 2017; Tang et al., 2018; Bain et al., 2019; Lim et al., 2019; Bethel et al., 2021). For example, the SUSTAIN-6 trial showed that the risk of retinopathy events was increased during the first 2 months of treatment with injectable semaglutide. Nevertheless, a *post hoc* analysis across the SUSTAIN trials (Marso et al., 2016a; Ahrén et al., 2017; Aroda et al., 2017; Sorli et al., 2017; Ahmann et al., 2018; Kaku et al., 2018; Pratley et al., 2018; Rodbard et al., 2018; Seino et al., 2018; Lingvay et al., 2019; Zinman et al., 2019b; Capehorn et al., 2020; Ji et al., 2020) associated the increased risk of diabetic retinopathy with being limited to the early stages of treatment and mainly caused by rapid improvements in glycemic control, *i.e.*, not a direct adverse effect of semaglutide (Vilsbøll et al., 2018). As part of the AngioSafe 1 study, both an observational and interventional phase IV study of patients with T2D showed that GLP-1R agonists did not increase the risk for severe diabetic retinopathy (Gaborit et al., 2020). This was further supported by another study comparing GLP-1R agonists to *thiazolidinedione* and *long-acting insulin* where GLP-1R agonists did not increase the risk of diabetic retinopathy over an average treatment time of less than 1 year (Wang et al., 2018). Accordingly, a cohort study found no association between the exposure to GLP-1R agonists and diabetic retinopathy, and in fact GLP-1R agonists were found to even decrease the risk of diabetic retinopathy by 33% when compared to new-users of insulin (Douros et al., 2018). Over a longer period of time, the progression of diabetic retinopathy after intensified glycemic control has also been demonstrated to be transient and reversible (Aiello and Dcct/Edic Research Group, 2014). The intensive glycemic control of diabetic patients appears to reduce both the risk of progression and the onset of diabetic retinopathy (Aiello and Dcct/Edic Research Group, 2014 with conventional antidiabetic treatments. Thus, the association between hypoglycemic agents and the exacerbation of diabetic retinopathy remains uncertain, and the early worsening is likely due to rapid lowering of blood glucose and intensified glycemic control, which, in the long run, also appears to be beneficial (Aiello and Dcct/Edic Research Group, 2014; Jingi et al., 2017; Tang et al., 2018; Vilsbøll et al., 2018; Wang et al., 2018; Bethel et al., 2021). A recent meta-regression analysis of GLP-1R agonist cardiovascular outcome trials (*LEADER, SUSTAIN-6, EXSCEL, HARMONY, REWIND*, and *PIONEER-6 trials*) has suggested that clinicians should consider the status of diabetic retinopathy in patients before initiating treatment with GLP-1R agonists (Bethel et al., 2021). In summary, the risk of diabetic retinopathy does not seem to be increased with the use of GLP-1R agonists and is being further investigated in ongoing trials (NCT02671864, NCT03811561). Thus, the patient’s retinopathy status must be determined before initiating treatment with incretin-based therapies as the GLP-1R agonists.

#### What Are the Possible Concerns About Glucagon-Like Peptide 1 Agonists as Potential Treatments for Glaucoma?

If GLP-1R agonists are to be used in the treatment of glaucoma patients, their retinopathy status must be determined before start of treatment. The most common type of glaucoma is primary open-angle glaucoma, with the typical patients being elderly and often underweight (Klein et al., 1992; Leske et al., 1994; Wensor et al., 1998; Jonasson et al., 2003; Iwase et al., 2004; de Voogd et al., 2005; Na et al., 2020). Accordingly, the appetite suppressant and weight loss effects of GLP-1R stimulation (Khoo and Lin, 2021) should be considered in potential trials with GLP-1R agonists as therapeutic agents for the treatment of glaucoma. Consideration should also be given as oral semaglutide has been associated with greater discontinuation of treatment in patient groups aged 65 years or older (Aroda et al., 2020). The most common reason stated for patients discontinuing oral semaglutide has been its mild to moderate gastrointestinal adverse effects, especially nausea, which is experienced by up to 50% of patients (Filippatos et al., 2014; Pratley et al., 2019; Zinman et al., 2019a; Aroda et al., 2020; Evaluate, 2020). Therefore, premature discontinuation of oral semaglutide due to adverse effects turn out to be only a few percent higher than its injectable alternatives. In the PIONEER-4 trial, 11% of the patients using oral semaglutide discontinued treatment because of adverse effects compared to 9% of the patients using subcutaneous liraglutide (Pratley et al., 2019). Another phase II interventional study found that premature cessation of treatment due to adverse effects was also higher for oral semaglutide than injectable semaglutide (Warren et al., 2018). However, this study as well as other studies (Davies et al., 2017; Warren et al., 2018; Aroda et al., 2020; Wright and Aroda, 2020) reported that both oral and injectable semaglutide were mostly discontinued due to gastrointestinal adverse effects. This together with the fact that patients showed treatment satisfaction in favor of injectable semaglutide rather than, e.g., injectable liraglutide in the SUSTAIN-10 trial (Capehorn et al., 2020), suggests that the higher proportion of patients discontinuing oral semaglutide treatment may be due to patients having to administer oral semaglutide once every day versus once a week with injectable semaglutide, making compliance for oral semaglutide more difficult and more expensive. Therefore, premature discontinuation of oral semaglutide may be due to the fact that elderly patients may be weaker, prefer treatment once a week, and are more susceptible to gastrointestinal intolerance (Newton, 2005) combined with baseline comorbidities that complicate continuation of treatment. Premature discontinuation of treatment due to adverse effects may also be simply due to the dosage of oral semaglutide and the rate of dose escalation. For instance, a dose of 7 mg oral semaglutide in the PIONEER 8 trial caused a lower percentage of patients discontinuing treatment compared to 14 mg oral semaglutide (7 mg: 9% and 14 mg: 13%, respectively) (Zinman et al., 2019a). Similar to the conventional subcutaneously administrated GLP-1R agonists, another study found that *slow* dose escalation of oral semaglutide resulted in a lower proportion of patients discontinuing treatment (Davies et al., 2017). Furthermore, the adverse effects of oral semaglutide tend to exacerbate as the dose is increased (Wright and Aroda, 2020). This suggests that the premature discontinuation of oral semaglutide in trials may also be due to patients’ expectations at the start of treatment and lack of counseling that effects such as nausea are frequent experiences that typically disappear over time. However, a recent Japanese health-related quality of life assessment of the PIONEER-10 trial showed that patients tended to be more satisfied when treated with oral semaglutide than with injectable dulaglutide (Ishii et al., 2021). Patients treated with oral semaglutide were also more adherent to the treatment (Ishii et al., 2021). This suggests that oral GLP-1 receptor agonists, compared to injectable GLP-1 receptor agonists, result in greater patient satisfaction and treatment persistence.

In summary, current knowledge about the potential disadvantages of GLP-1R agonist drugs is few compared to the potential beneficial neuroprotective properties. Semaglutide, in both injectable and oral formulations, appears to be a promising potential GLP-1R agonist in combination with conventional IOP-lowering agents to decrease the rate of glaucoma worsening. However, it would be wise to assess retinopathy status, patients’ BMI, age and preference for weekly subcutaneous injections or daily oral administrations as well as the dose before starting treatment.

### Glucagon-Like Peptide 1 Agonists Show Neuroprotective Properties in the Treatment of Other Neurodegenerative Diseases

**FIGURE 3:**
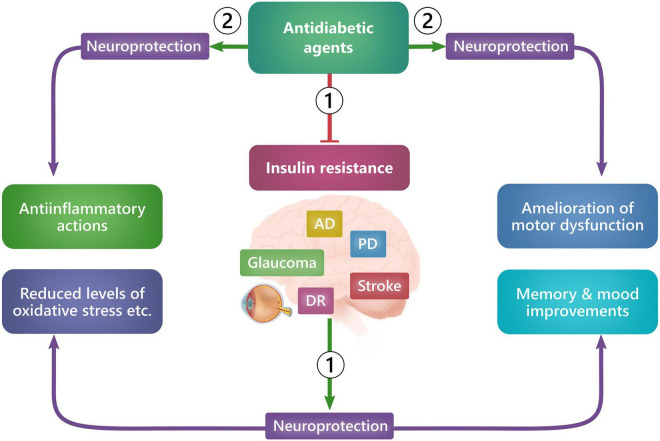
Ways in which GLP-1R agonists exert neuroprotection. GLP-1R agonists can exert neuroprotection either systemically (1), by improving glycemic control and reducing insulin resistance, or locally (2), by acting on receptors in neuronal tissue cells and thus causing, e.g., antiinflammation, preservation of memory, dopaminergic neurons, motor function and mood improvements. *AD: Alzheimer’s disease, DR: Diabetic retinopathy, PD: Parkinson’s disease*.

Glucagon-like peptide 1 (GLP-1) and GLP1R agonists have been associated not only with neuroprotection in the retina but also in the brain in various animal models of neurodegenerative diseases (Zhang et al., 2018, 2019; Basalay et al., 2019; Yang et al., 2019; Chang et al., 2020; Zhai et al., 2020). The promising preclinical results have now paved the way for evaluating the use of GLP-1R agonists in clinical studies with neurodegenerative diseases such as *Alzheimer’s disease*, *Parkinson’s disease*, *stroke*, and *diabetic neuropathy*. The neuroprotective effect of GLP-1R agonists is that it can either directly/locally cause neuroprotection by acting on neuronal tissue or indirectly/systemically cause neuroprotection by improving glycemic control and treating existing insulin resistance (Figure 3) (Fiory et al., 2019; Femminella et al., 2021).

#### Alzheimer’s Disease

Several early studies have identified the presence of cognitive impairment in patients with T2D (Perlmuter et al., 1984; Helkala et al., 1995; Vanhanen et al., 1999), and T2D has been recognized as a risk factor for the development of Alzheimer’s disease (Janson et al., 2004). In addition, Alzheimer’s disease has also been directly linked to insulin resistance (Neth and Craft, 2017; Arnold et al., 2018; Hölscher, 2019; Kellar and Craft, 2020), even in the absence of concomitant diabetes (Schrijvers et al., 2010). In this case, peripheral insulin resistance was associated with decreased hippocampal glucose metabolism and lower volume of gray matter in a population of non-diabetic patients with Alzheimer’s disease (Femminella et al., 2021). Similarly, patients with cognitive impairment and Alzheimer’s disease have been shown to improve in memory, attention, mental tasks, cognition, and brain activation following insulin treatment, both intravenously and intranasally (Craft et al., 1996, 1999, 2003, 2012, 2017; Kern et al., 2001; Reger et al., 2006, 2008a,b; Claxton et al., 2015; Femminella et al., 2021; Hallschmid, 2021). Although these are promising results, evidence for a protective role for insulin remains low, and a recent Cochrane review from 2017 found no protective or preventive association between cognitive impairment and the use of antidiabetic T2D treatment strategies (Areosa Sastre et al., 2017) (i.e., *metformin, insulin, repaglinide, glibenclamide, glimepiride, rosiglitazone, gliclazide, perindopril-indapamide*). An aspiring novel take on insulin regulation is the administration of GLP-1R agonists, as these both regulate insulin sensitivity in the brain (Sandoval and Sisley, 2015) and possibly exert direct neuroprotective effects (Figure 3).

Besides preclinical studies pointing toward a potential use of GLP-1R agonists in the treatment of Alzheimer’s (McClean et al., 2011; Chang et al., 2020), a recent *post hoc* analysis led by Novo Nordisk comprising 15,820 patients with T2D, the use of the GLP-1R agonists *liraglutide* and *semaglutide* was also associated with a 53% significantly lower risk of developing dementia (Ballard et al., 2020). These findings have led to the initiation of the EVOKE trial program in 2021, which will enlighten a potential neuroprotective effect of oral semaglutide in the future. Previously, a neuroprotective effect of GLP-1R agonists has also been supported by long-term treatment with another GLP-1R agonist, *dulaglutide*, which reduced cognitive impairment in patients with T2D compared to placebo (NCT01394952; *the REWIND trial*) (Cukierman-Yaffe et al., 2020). 6-months of treatment with the GLP-1R agonist *liraglutide* (Victoza^®^) in Alzheimer’s disease patients was found to prevent declines in cerebral glucose metabolism, which is associated with synaptic dysfunction, cognitive impairment, and progression of the disease (Gejl et al., 2016) (Table 5). 12 weeks liraglutide-treatment of patients at high risk for dementia was also shown to increase connectivity in the default mode network supporting the fact that GLP-1R agonists may reduce or hinder the progression of Alzheimer’s disease (Watson et al., 2019). Supporting the hypothesis that increased GLP-1R signaling potentially promotes neuroprotection, DPP4-inhibitors are also associated with improved cognitive function (Isik et al., 2017; Xue et al., 2020). The use of GLP-1R agonists in Alzheimer’s disease also appears to be beneficial in an as yet unpublished multicenter, 12-month, phase II UK trial (NCT01843075, *the ELAD-study*), which evaluates liraglutide as a potential therapeutic agent in Alzheimer’s patients with mild dementia (Femminella et al., 2019). Results from the ELAD-study, presented at the Clinical Trials on Alzheimer’s Disease Conference (CTAD) 2020, showed that daily injections of liraglutide improved secondary outcomes of the study, *i.e.*, the volume of gray matter and the cognitive function measured as improvements in Alzheimer’s Disease Assessment Scale (ADAS) scores (Evaluate, 2020). Thus, the use of GLP-1R agonists and DPP4-inhibitors has been reported to prevent cognitive impairment in several clinical studies (Gejl et al., 2016; Isik et al., 2017; Mansur et al., 2017; Watson et al., 2019; Ballard et al., 2020; Cukierman-Yaffe et al., 2020; Xue et al., 2020).

**TABLE 5:** Clinical studies elucidating the use of GLP-1R agonists in Alzheimer’s disease and cognitive dysfunction in other conditions.

Compound		Study types	Study design	Study outcome	References
Alzheimer’s Disease (AD)	Exenatide	Interventional pilot study	18 patients with high-probability AD were randomly assigned to receive exenatide or placebo. However, the study was finalized before time due to withdrawal of sponsor support, which was not related to safety considerations.	Possibly decreased Aβ42-levels.	Mullins et al., 2019
	Liraglutide	Phase II interventional	81 patients with AD or subjective cognitive complaints were randomly assigned to receive liraglutide or placebo for 12 weeks, 26 weeks or 12 months.	Improved the volume of gray matter and enhanced the ADAS-Exec z-score. Prevented decline in cerebral glucose metabolic rate and improved connectivity in several brain regions, i.a. the default mode network.	Gejl et al., 2016; Watson et al., 2019
Mood Disorders	Liraglutide	Interventional	19 patients with major depressive or bipolar disorder and impaired executive function were treated with liraglutide (1.8 mg/day) as an add-on to existing medication.	Improved the cognitive function, and response to treatment was better in individuals with higher baseline insulin resistance and BMI.	Mansur et al., 2017
Diabetes	DPP4-inhibitors (e.g., sitagliptin)	Phase N/A interventional, observational	265 patients with T2D with/without post-stroke mild cognitive impairment (MCI) were randomly assigned to receive DPP4-inhibitors. Patients with T2D using sitagliptin were observed.	Improved cognitive ability in post-stroke MCI patients and cognitive function in patients with and without AD.	Isik et al., 2017; Xue et al., 2020
	Dulaglutide	Phase III interventional	9,901 patients with T2D were randomly assigned to receive dulaglutide and followed up at least every 6 months.	Reduced hazard of cognitive impairment by 14%.	Cukierman-Yaffe et al., 2020

*The use of agents increasing GLP-1 receptor signaling may be associated with a neuroprotective effect in Alzheimer’s disease (AD) by preventing declines in cortical activity, decreasing Aβ-levels and improving cognitive function as well as the volume of gray matter of patients. T2D; Type 2 diabetes.*

In contrast, a recent pilot study could not conclude any neuroprotective property of the GLP-1R agonist exenatide, other than a reduced level of Aβ (NCT01255163) (Mullins et al., 2019). However, the lack of significant results favoring a neuroprotective effect of GLP-1R agonists may be due to early business-related withdrawal of sponsor support (Mullins et al., 2019). Thus, there is evidence in favor of a neuroprotective effect of GLP-1R agonists in Alzheimer’s disease, which will hopefully be better elucidated in ongoing clinical trials such as the EVOKE trial program.

#### Parkinson’s Disease

Clinical studies on GLP-1R agonists in Parkinson’s disease have focused on the GLP-1R agonist *exenatide*, which has yielded many promising results (Aviles-Olmos et al., 2013, 2014; Athauda et al., 2017, 2018, 2019a,b; Brauer et al., 2020) (Table 6). These notable developments have even led to the initiation of a phase III clinical trial with exenatide (Bydureon^®^) as an antiparkinsonian agent, launched in the beginning of 2020 (NCT04232969). The observed neuroprotective effects of GLP-1R agonists can be attributed to a possible mechanistic link between Parkinson’s disease and diabetes. Low insulin sensitivity has, similar to Alzheimer’s disease, been associated with an increased risk of developing Parkinson’s disease (Athauda and Foltynie, 2016). In addition, insulin is expected to have a decisive influence on the dopaminergic system (Fiory et al., 2019). Death of dopaminergic neurons in the substantia nigra pars compacta and changes in striatal nuclei in patients with Parkinson’s disease have been correlated with remarkable changes in structures involved with insulin signaling (Fiory et al., 2019). A possible mechanism behind the properties of exenatide in the study by Athauda et al. (2019a) was suggested to be a normalization of brain insulin signaling and an involvement of Akt signaling pathways. This was supported through electrochemiluminescence assay quantifications of proteins related to insulin signaling (i.e., phosphorylated forms of Akt) from neuronal-derived exosomes harvested from peripheral blood samples of Parkinson’s patients who had participated in the study (NCT01971242) (Athauda et al., 2019a).

**TABLE 6:** Clinical studies elucidating the use of GLP-1 receptor agonists in Parkinson’s disease.

Compound		Study types	Study design	Study outcome	References
Parkinson’s Disease (PD)	Exenatide	Phase II interventional	107 patients with PD were randomly assigned to receive exenatide for 48 weeks or 12 months.	Improved motor and cognitive symptoms of patients, which persisted even 12 months after last exenatide-exposure.	Aviles-Olmos et al., 2013, 2014; Athauda et al., 2017
		*Post hoc* analyses	*Post hoc* analyses of studies assessing the motor and non-motor symptoms, e.g., the cognitive function, mood and emotional well-being of 60 patients with PD treated with exenatide.	Improved motor and non-motor symptoms of patients included in the analyses. Patients with older age and PD duration over 10 years responded less well to treatment with exenatide.	Athauda et al., 2018, 2019a
	Incretin-based therapies*	Observational	Retrospective cohort and nationwide case-control study assessing the incidence of PD among 106,168 patients with T2D treated with, e.g., DPP4-inhibitors and/or GLP-1 receptor agonists.	Reduced the incidence of PD, even when patients were exposed to incretin-based therapies for a short period of time (up to 12 and 12-36 months).	Svenningsson et al., 2016; Brauer et al., 2020

*Clinical studies on the use of GLP-1 receptor agonists in patients with PD have focused on exenatide, and there remains a further need to investigate the neuroprotective effects of DPP4-inhibitors. Off note, exenatide has been associated with improvements in motor and non-motor symptoms of PD patients.*

*Incretin-based therapies include: DPP4-inhibitors and GLP-1R agonists.

A recent population-based cohort study by Brauer et al. (2020) also found evidence of a lower incidence of Parkinson’s disease in diabetic short-term and long-term users of GLP-1R agonists and DPP4-inhibitors (Brauer et al., 2020), suggesting beneficial assets of anti-diabetic drugs in the fight against Parkinson’s disease. This finding has been confirmed by other studies in which subcutaneous administration of exenatide to randomly assigned Parkinson’s patients was associated with greatly improved outcome measures that assessed the severity of motor symptom (Aviles-Olmos et al., 2013, 2014; Athauda et al., 2017, 2018). Motor function of Parkinson’s patients who have previously been exposed to exenatide has been shown to improve even 12 months after terminated treatment (Aviles-Olmos et al., 2014). Furthermore, exenatide appears to both suppress motor impairments (*i.e.*, dyskinesia) and improve cognitive function of patients with Parkinson’s disease (Aviles-Olmos et al., 2013; Athauda et al., 2017). In addition, a *post hoc* analysis further revealed that exenatide improved a wide range of non-motor symptoms (*i.e.*, mood, depression) in patients with Parkinson’s disease compared to placebo (Athauda et al., 2018). Concerning the treatment of Parkinson’s patients with GLP-1R agonists, there seems to be one compelling aspect regarding effectiveness of the treatment, and that is that the agonists should be administered as early as possible after diagnosis, as increasing age and disease duration over 10 years is associated with less effective response to exenatide treatment (Athauda et al., 2019b).

The effects of other GLP-1R agonists in the treatment of patients with Parkinson’s disease are also in line to be elucidated. A phase II study is currently investigating the neuroprotective effects of injectable *semaglutide* in 270 Parkinson’s patients in a double-blinded, placebo-controlled design (NCT03659682). Also, *liraglutide* (NCT02953665), *lixisenatide* (NCT03439943), *exenatide* (NCT04305002), sustained release exenatide – *PT320* (NCT04269642) and a pegylated form of exenatide, *NLY01* (NCT04154072), are all undergoing clinical investigation in Parkinson’s disease.

While GLP-1R agonists show promising results, there is a need to further investigate the neuroprotective effects of DPP4-inhibitors in Parkinson’s patients (Svenningsson et al., 2016; Brauer et al., 2020; Ece Bayram and Irene Litvan, 2020). Nevertheless, the numerous previous studies of exenatide combined with the initiation of a phase III clinical trial of exenatide, combined with ongoing trials, undeniably provide a promising basis for the prospective neuroprotective use of GLP-1R agonists in Parkinson’s disease.

#### Stroke

Stroke is associated with cognitive dysfunction (O’Brien et al., 2003), glaucoma (Lee et al., 2018; Rim et al., 2018), and secodary neurodegeneration (Ong et al., 2017). Several preclinical studies have observed GLP-1R agonists as neuroprotective compounds in rodent stroke models (Basalay et al., 2019; Yang et al., 2019), and GLP1-R agonists have been suggested for the use in patients with ischemic stroke (Maskery et al., 2021). In general, studies on the potential of GLP-1R agonists in stroke have focused on its effectiveness in either treating acute stroke or preventing the onset of stroke. In acute stroke, GLP-1R agonists have been suggested to treat stroke-induced hyperglycemia (*post-stroke hyperglycemia*) that is associated with, e.g., disruption of the blood-brain-barrier, intensified inflammatory reactions, edema, increased infarct size and worsening of functional outcomes (Li et al., 2013). Compared to insulin, GLP-1R agonists also have the advantage of preventing incidences of hypoglycemia. In a pilot study, 11 patients with ischemic stroke were treated with exenatide within app. 4-12 h after onset appeared to reduce hyperglycemia and hypoglycemic incidences (Daly et al., 2013). Another ongoing study (TEXAIS-study) also assesses the potential of exenatide in acute stroke (NCT03287076) (Muller et al., 2018). GLP-1R agonists have also shown effect in preventing stroke. Meta-analyses of large cardiovascular outcome trials have demonstrated that GLP-1R agonists, i.e., *dulaglutide*, *liraglutide*, *injectable* and *oral semaglutide*, have a potential stroke-protective effect (Pfeffer et al., 2015; Marso et al., 2016a,b; Holman et al., 2017; Hernandez et al., 2018; Kristensen et al., 2019; Alfayez et al., 2020; Gerstein et al., 2020; Strain et al., 2020). Another meta-analysis of large cardiovascular outcome trials such as SUSTAIN (*injectable semaglutide*), LEADER (*liraglutide*), HARMONY (*albiglutide*) (Hernandez et al., 2018), ELIXA (*lixisenatide*) (Pfeffer et al., 2015) and EXSCEL (*exenatide*) (Holman et al., 2017) appeared to reduce the risk of total stroke (by, *i.e.*, 13% or 16%) (Bellastella et al., 2020; Gerstein et al., 2020). Semaglutide and dulaglutide were also associated with significantly reduced risks for stroke, where semaglutide appear to be better (Alfayez et al., 2020; Evans et al., 2021). However, in line with the potential neuroprotective effect of GLP-1R agonists in ischemic stroke, Rigshospitalet Denmark is running a randomized clinical trial of exenatide as an organ protecting agent, e.g., a brain-protecting agent, where stroke is one of the primary outcome measures (NCT02673931). Another Danish study is also investigating the effect of GLP-1R agonists on cerebral blood flow (NCT02829502). In fact, there are many other recruiting or completed studies investigating the protective effects of GLP-1R agonists (NCT02838589, NCT00418288, NCT00256256, NCT03948347) and also DPP4-inhibitors (NCT01107886, NCT00968708) in stroke. Thus, GLP-1R agonists show encouraging potential as stroke-protective agents, but there is still a need to investigate their effects in further clinical therapeutic trials.

#### Diabetic Neuropathy

Several preclinical studies have suggested that GLP-1 receptor activation is a promising mechanism that prevents neurodegeneration and improves neuroprotection in peripheral nerves (Yamamoto et al., 2002; Perry et al., 2007; Luciani et al., 2010; Griffioen et al., 2011; Himeno et al., 2011; Kan et al., 2012). A prospective, open-label pilot study of the DPP4-inhibitor *teneligliptin* in patients with T2D treatment has been shown to improve the patient’s peripheral and autonomic neuropathy status (Syngle et al., 2021). An unpublished clinical study has also associated a lacking responsiveness to GLP-1R agonist treatment (*liraglutide* and *exenatide*) with a higher presence of cardiovascular autonomic neuropathy (*Clinical Trial Registration Number: 7459*) (Duvnajak et al., 2017). However, DPP4-inhibitors, *e.g., saxagliptin*, have shown to have a neutral impact on the incidence of diabetic neuropathy (Taylor and Lam, 2020). A proof-of concept open-label randomized clinical trial has also assessed the effect of GLP-1R agonists on 46 patients with T2D and mild to moderate diabetic peripheral neuropathy (Jaiswal et al., 2015). Patients were randomized to be treated with either exenatide or insulin glargine for 18 months. The trial found no statistically significant effect on measures of neuropathy, *i.e.*, no significant differences in confirmed clinical neuropathy, measures of cardiovascular autonomic neuropathy, nerve conduction tests nor intra-epidermal nerve fiber densities (Jaiswal et al., 2015). Although, the lack of significant differences in patients’ neuropathic statuses might be the result of: (1) the comparison to insulin, which also promotes glycemic control, and (2) that GLP-1 receptor agonists mainly prevent the development of neuropathy. The latter is also supported by the TODINELI-trial (Brock et al., 2019), where treatment of type 1 diabetic patients with liraglutide reduced levels of proinflammatory cytokines (IL-6) but did not improve the status of patients that had already established autonomic or diabetic polyneuropathy. Thus, clinical trials related to the neuroprotective role of GLP-1R agonists and DPP4-inhibitors in neuropathy are sparse (Mehta et al., 2021). Nevertheless, promising results from preclinical trials and the TODINELI-trial promote the idea of investigating the protective effects of GLP-1R agonists on neuropathy in further clinical trials.

## Conclusion

The present review has summarized the current clinical evidence for a potential use of GLP-1R agonists in prospective neuroprotective treatment strategies against glaucoma and other neurodegenerative diseases. GLP-1R agonists have exerted a neuroprotective effect in several preclinical studies in both the brain and the retina. The most promising GLP-1R agonist in terms of efficacy, adverse effects, convenience for patients, cost-effectiveness and risk of cardiovascular complications is semaglutide, which is the only GLP-1R agonist that can be administrated as both an oral and injectable formulation. A recent association between exposure to GLP-1R agonists and a reduced risk of glaucoma makes it even more justified to consider the neuroprotective potential of GLP-1R agonists, particularly semaglutide, in anti-glaucomatous treatment strategies. Agents that increase GLP-1R signaling also appear to reduce the likelihood of developing diabetic retinopathy, AMD, cognitive dysfunction, motor dysfunction, stroke-induced neurodegenerative impairments, and neuropathy, supporting a potential neuroprotective effect of semaglutide in glaucoma. Furthermore, as oral semaglutide has recently entered phase III trials against Alzheimer’s disease, and injectable semaglutide is in the phase II trial of Parkinson’s disease as well as in the phase III trial against diabetic eye diseases, the potential of specifically semaglutide in glaucoma is additionally endorsed. However, there are currently no clinical nor any preclinical studies assessing the neuroprotective effects of semaglutide in glaucoma. Therefore, future studies are strongly needed to further investigate the potential of semaglutide as a repurposed novel neuroprotective agent in the treatment of glaucoma.

## Author Contributions

All authors listed have made a substantial, direct, and intellectual contribution to the work, and approved it for publication.

## Conflict of Interest

The authors declare that the research was conducted in the absence of any commercial or financial relationships that could be construed as a potential conflict of interest.

## Publisher’s Note

All claims expressed in this article are solely those of the authors and do not necessarily represent those of their affiliated organizations, or those of the publisher, the editors and the reviewers. Any product that may be evaluated in this article, or claim that may be made by its manufacturer, is not guaranteed or endorsed by the publisher.
